# Genome Sequencing and Comparative Transcriptomics Provide a Holistic View of 4-Nitrophenol Degradation and Concurrent Fatty Acid Catabolism by *Rhodococcus* sp. Strain BUPNP1

**DOI:** 10.3389/fmicb.2018.03209

**Published:** 2019-01-04

**Authors:** Kriti Sengupta, Martin T. Swain, Paul G. Livingstone, David E. Whitworth, Pradipta Saha

**Affiliations:** ^1^Department of Microbiology, Burdwan University, Bardhaman, India; ^2^Institute of Biological, Environmental and Rural Sciences, Aberystwyth University, Aberystwyth, United Kingdom

**Keywords:** 4-nitrophenol, *Rhodococcus*, biodegradation, genome sequence, transcriptome

## Abstract

*Rhodococcus* sp.strain BUPNP1 can utilize the priority environmental pollutant 4-nitrophenol (4-NP) as its sole source of carbon and energy. In this study, genome and transcriptome sequencing were used to gain mechanistic insights into 4-NP degradation. The draft BUPNP1 genome is 5.56 Mbp and encodes 4,963 proteins, which are significantly enriched in hypothetical proteins compared to other *Rhodococcus* sp. A novel 4-NP catabolic 43 gene cluster “*nph*” was identified that encodes all the genes required for the conversion of 4-NP into acetyl-CoA and succinate, via 4-nitrocatechol. The cluster also encodes pathways for the catabolism of other diverse aromatic compounds. Comparisons between BUPN1 growing on either 4-NP or glucose resulted in significant changes in the expression of many *nph* cluster genes, and, during 4-NP growth, a loss of lipid inclusions. Moreover, fatty acid degradation/synthesis genes were found within the *nph* cluster, suggesting fatty acids may be concurrently catabolised with 4-NP. A holistic model for the action of the *nph* gene cluster is proposed which incorporates genetic architecture, uptake and metabolism of aromatic compounds, enzymatic activities and transcriptional regulation. The model provides testable hypotheses for further biochemical investigations into the genes of the *nph* cluster, for potential exploitation in bioremediation.

## Introduction

The Environmental Protection Agency (EPA) of the United States has declared 4-nitrophenol (4-NP) a priority pollutant (Keith and Telliard, 1979). 4-NP is a nitro-aromatic compound used in various chemical industries to manufacture dyes, explosives, herbicides/pesticides, and pharmaceuticals. It is moderately water soluble and released into agricultural soils by the microbial degradation of pesticides like parathion, methyl parathion and other agrochemicals (Heitkamp et al., 1990). Due to its widespread occurrence, prolonged retention time in ecosystems and toxicity, the effective removal of 4-NP from contaminated sites is of huge public concern and interest. Bioremediation using microbial cells offers an effective alternative to costly and technologically demanding physical or chemical methods (Kou-San and Parales, 2010). Microbial degradation is the main route for the removal of 4-NP from the environment and numerous bacteria have been reported to degrade it (Barik and Sethunathan, 1978; Swindoll et al., 1988).

Two major pathways for 4-NP biodegradation into intermediates of central metabolism have been characterized so far. One, leading to the formation of 4-nitrocatechol (4-NC) and benzene 1,2,4-triol (BT, also known as hydroxyquinol), has been reported in Gram-positive bacteria, such as *Arthrobacter* spp., *Bacillus* spp., and *Rhodococcus* spp. (Kadiyala and Spain, 1998; Kitagawa et al., 2004; Chauhan et al., 2010; Sengupta and Saha, 2014; Sengupta et al., 2016). The second, leading to formation of a hydroquinone (HQ) intermediate, has been reported in Gram-negative bacteria, such as *Burkholderia* spp., *Moraxella* spp., and *Pseudomonas* spp. (Spain and Gibson, 1991; Chauhan et al., 2000; Zhang et al., 2009). Among degrading bacteria, environmental strains of *Rhodococcus* genus are of particular interest due to their astonishing metabolic diversity. They have been shown to exhibit a broad range of enzyme activities which permit them to degrade diverse classes of nitro-aromatic compounds (Bell et al., 1998; Larkin et al., 2005; Ghosh et al., 2010) into acetyl CoA and succinyl CoA, which feed into central metabolism (Harwood and Parales, 1996; Fuchs et al., 2011; Sainsbury et al., 2013). However, relatively few studies have investigated the genetic basis of such activities (Chauhan et al., 2000; Kitagawa et al., 2004; Takeo et al., 2008; Yamamoto et al., 2011), instead focusing on identification of degradation intermediates.

Currently, genome sequence information is only available for a few aromatic-degrading members of the *Rhodococcus* genus. These include *Rhodococcus* sp. strain PN1, *Rhodococcus erythropolis* PR4, *Rhodococcus imtechensis* RKJ300, *Rhodococcus jostii* RHA1, and *Rhodococcus opacus* SAO101, which have been reported to be able to degrade 4-NP (Kitagawa et al., 2004; McLeod et al., 2006; Takeo et al., 2008; Yamamoto et al., 2011; Vikram et al., 2013). Systematic studies addressing transcriptome changes in the presence of 4-NP are absent from the literature, although for strain PN1, two 4-NP catabolism gene clusters have been identified experimentally (Takeo et al., 2008; Yamamoto et al., 2011).

In order to rationally develop a useful sustainable strategy for 4-NP bioremediation, it is important to understand the molecular mechanisms of adaptation employed by 4-NP degrading bacteria. In this study, we employed genome sequencing and transcriptome profiling to identify the genes involved in 4-NP degradation in an environmental *Rhodococcus sp*. strain BUPNP1 (Sengupta and Saha, 2014). After sequencing the genome of BUPNP1 we compared the transcriptomes of cells grown in the presence of 4-NP to that of cells grown with glucose, in order to identify the transcriptional changes associated with exposure to 4-NP. A large cluster of genes was identified which encoded the enzymes necessary for the complete degradation of 4-NP into acetyl CoA via 4-NC, distributed amongst genes encoding enzymes for the degradation of other aromatic compounds, including phenol, benzoate and halobenzoates. Many genes proposed to be involved in 4-NP degradation were differentially expressed on 4-NP exposure.

We propose an integrated model of 4-NP degradation by BUPNP1, reconciling genetic structure with transcriptional regulation, enzymatic activity, chemical intermediates and metabolic pathways. This model provides a framework for further characterization of the enzymes and pathways of 4-NP degradation, directed at developing a successful bioremediation platform for the efficient removal of toxic 4-NP contamination.

## Materials and Methods

### Genome Sequence Analysis

Genomic DNA from strain BUPNP1 was extracted and sequenced by MicrobesNG (Birmingham, UK) using the Illumina HiSeq platform (250 bp paired end protocol). Genomic DNA libraries were prepared using a Nextera XT Library Prep Kit (Illumina, San Diego, USA) following the manufacturer's instructions with two modifications: two nanograms of DNA instead of one were used as input, and the PCR elongation time was increased to 1 min from 30 s. Pooled libraries were quantified using the Kapa Biosystems Library Quantification Kit for Illumina on a Roche light cycler 96 qPCR machine. Libraries were sequenced and the reads were adapter trimmed using Trimmomatic 0.30 with a sliding window quality cutoff of Q15 (Bolger et al., 2014). *De novo* assembly was performed on samples using SPAdes version 3.7 (Bankevich et al., 2012), and contigs were annotated using Prokka 1.11 (Seemann, 2014) and submitted to RAST for further annotation (Supplemental File 1). A circular view of the genome was obtained using BASys (Van Domselaar et al., 2005). The presence of plasmids was tested using PlasmidFinder (Carattoli et al., 2014), family membership of regulatory proteins was obtained using P2RP (Barakat et al., 2013), while clusters of orthologous groups (COG) analysis was performed by WebMGA (Wu et al., 2011). Relative abundances of each COG present in BUPNP1 was evaluated by one-sample *t*-test with the other 4-NP degrading genomes of *Rhodococcus* sp. (*R. erythropolis* PR4, *R. imtechensis* RKJ300, *R. jostii* RHA1, and *R. opacus* B4) which had been re-annotated using the same pipeline as for BUPNP1. Average nucleotide identity (ANI) values were calculated using Kostas lab-ANI Calculator (Goris et al., 2007). For the creation of Figure 3, genome nucleotide sequences were aligned using NUCmer version 3.07 from the MUMmer package (Delcher et al., 2003), with the minmatch parameter set to 10 base-pairs. Each genome sequence was aligned in a pairwise manner to *Rhodococcus jostii* RHA1 (NC_008268). From the same package, the show-coords program was used to output the coordinates of each aligned fragment. These coordinates were then formatted according to the requirements of the circos visualization package (Krzywinski et al., 2009).

### Culture Conditions

For inoculum preparation, BUPNP1 was grown in TSB (Tryptic soya broth; constituents in w/v: 1.7% tryptone, 0.5% soybean casein digest, 0.5% NaCl, 0.25% dextrose, 0.25% K_2_HPO_4_, pH 7.3) and the cell mass obtained was washed thrice with phosphate buffer saline (137 mM NaCl, 2.7 mM KCl, and 10 mM phosphate buffer, pH 7.4). The resulting cell mass was used to inoculate Minimum Mineral salt media (MM) at pH 7.2 (Prakash et al., 1996) supplemented with either 0.5 mM 4-NP, 0.55 mM glucose, 0.3 mM 4-NC, or 0.25 mM BT as sole sources of carbon and energy. Cultures were incubated in a rotary shaker (120 rpm) at 35°C. Growth was monitored by measuring the increasing optical density at 600 nm (OD_600_) as well as viable cell counts by dilution plating onto fresh MM plates containing 4-NP. Optimization of substrate concentration, pH and temperature was performed for maximum growth and biodegradation of 4-NP.

### BIOLOG Assays

Carbon substrate utilization profiles of BUPNP1 were obtained using a VITEK 2 (BioMérieux, Inc., Hazelwood, MO, USA) with BCL cards (for Gram positive spore-forming bacilli) as per manufacturer's instruction. Each well of a BCL card was inoculated with 150 μl of a cell suspension of BUPNP1 (grown in TSB with an optical density at 600 nm of 0.6), loaded into a cassette and incubated at 35.5 ± 1°C. Development of color in the wells was monitored at 12 and 24 h and on the basis of the results obtained for 46 biochemicals tests, a catabolic profile was generated for the strain.

### Biodegradation of 4-NP and Pathway Intermediate Identification

BUPNP1 cell density and the concentration of 4-NP in MM were monitored using a UV–Vis spectrophotometer (Carry 50, Varion) at 600 and 400 nm, respectively every 6 h. The release of nitrite was determined periodically at 540 nm every 6 h using the method of White et al. (1996). Residual aromatic compounds were extracted from the culture supernatant using ethyl acetate (Ghosh et al., 2010), drying over anhydrous sodium sulfate and evaporating to dryness using a rotary evaporator. The dried extract was dissolved in 1 ml of acetonitrile and subjected to HPLC which was performed on a HiQ sil C_18_ column (250 mm X 4.6 ID) with UV/Vis absorbance detector and SHIMAZDU HPLC pump (SPD-M20A). A gradient of acetonitrile: water (80:20–0:100) was used as the mobile phase at a flow rate of 1 ml/min with a UV–Vis detector monitoring at 310 nm. Identification of the compounds was carried out by comparing retention time (RT) of the test samples to that of authentic reference standards. A time-course 4-NP monooxygenase assay was also performed as described by Ghosh et al. (2010) against a control lacking 4-NP substrate.

### TEM Visualization

Visualization of cross-sections through BUPNP1 cells was performed using a transmission electron microscope (TEM) for cells grown in culture media having either 4-NP or glucose as carbon source. After 12 h of incubation cells were harvested from each condition and samples prepared as previously described (Livingstone et al., 2018).

### Lipid Assays

The presence of lipid was quantified by the lipid specific dye, Nile red, followed by cell sorting by using a flow cytometer, based on the method of Blazeck et al. (2014). Flow cytometric analysis was performed using a Sony SH800 flow cytometer with cell sorting system, using filters for laser excitation at 488 nm and emission detection at 575 nm. At least 5,000 events were collected for each measurement, with gating by forward and backward scattering. The lipid contents of 4-NP and glucose grown cells were compared and expressed as an average change in Nile red fluorescence. In addition, lipid-containing cells (at least 10 million cells) were sorted and visualized with a confocal laser scanning microscope (Leica, TCS SP5).

### RNA Sequencing (RNA-seq) and Analysis

Total RNA was extracted from cell masses obtained from triplicated 6 and 12 h cultures in MM supplemented with either 0.5 mM 4-NP (test) or 0.55 mM glucose (control). RNA was extracted by using the TRIzol method (Chomczynski and Sacchi, 1987) for bacterial cells with a modification to include a mixing step with the addition of 100 mg of ultrafine glass beads at the TRIzol buffer step to break open the tougher Gram-positive cells. Following this, 2 μg of total RNA was processed using the Illumina Ribo-Zero Gold (Bacteria) kit to remove ribosomal RNA and the samples were prepared for sequencing using the Illumina TruSeq Stranded mRNA library kit (skipping the poly-A enrichment step). Each sample was uniquely indexed and sequenced on an Illumina HiSeq2500 platform in 2 × 126 bp read format. Transcriptome analysis was performed using RSEM version 1.2.26. Following a standard protocol for RSEM, coding sequences were extracted from the assembled genome using Prokka gene annotations, and prepared as a set of reference sequences for the RSEM mappings (rsem-prepare-reference). Quality control and adaptor trimming was performed on the RNA-Seq reads using Trimmomatic. RSEM was used to perform the following operations: first the gene expression values for each sample were calculated using bowtie2 (rsem-calculate-expression), these include the fragment per kilobase per million mapped reads (FPKM) metric that denote the counts of cDNA fragments originating from each gene, thus deriving an expression profile for each replicate. The gene expression values were formatted into a data matrix (rsem-generate-data-matrix), and then used by EBSeq to calculate the differential expression values and fold-changes for each experiment (rsem-run-ebseq), including the posterior probability of being differentially expressed (PPDE). Controlling for the false discovery rate (FDR) was performed using rsem-control-fdr with a hard threshold for the FDR rate equal to 0.05. Finally, an in-house script was used to calculate the log_2_ fold-changes, and to collate the FPKM values into a single table. Multivariate analysis of the datasets was undertaken using the EdgeR package in R.2.11.1 (Robinson et al., 2010).

## Results

Strain BUPNP1 was originally isolated from a landfill site in Burdwan, India. It was able to grow on 4-NP as sole carbon source and was initially identified as belonging to *Rhodococcus* sp. based on its general biochemical characteristics and 16S rDNA phylogeny (Sengupta and Saha, 2014). BUPNP1 is able to remove 4-NP efficiently from its medium, with the highest 4-NP monooxygenase specific activity yet recorded for a *Rhodococcus* strain (Supplemental File 2). In order to identify genes responsible for 4-NP degradation, initially the genome of BPNP1 was sequenced as a prerequisite for transcriptome analysis.

### The Genome of BUPNP1 Is Similar to That of Other 4-NP Degrading *Rhodococcus* sp.

BUPNP1 genomic DNA was sequenced and assembled into a draft genome of 5,555,112 bp, with 68.1% GC content. The general features of the BUPNP1 genome and those of other close species are presented in Table 1. It comprised 89 contigs (of length >200 bp) with the largest being 446,704 bp in length. The assembled genome had an N50 value of 192,622 bp and an L50 value of 10 (the longest 10 contigs together constituted half of the total sequence length, with the 10th longest contig having a size of 192,622 bp). While the genomes of some toxicant degrading *Rhodococcus* sp. have been reported to possess plasmid sequences encoding aromatic degrading genes (Na et al., 2005; Sekine et al., 2006; Takeda et al., 2010), strain BUPNP1 did not contain any plasmids. A total of 5,033 coding DNA sequences (CDS) were annotated, among which 4,963 were protein coding genes, with 54 tRNA, 9 rRNA, 3 ncRNA, and 70 pseudogenes. Of the protein coding genes, 4,489 (90.4%) were assigned to COG functional categories.

**Table 1:** Comparison of the genomic features of the BUPNP1 genome with its closest relatives.

**General features**	***Rhodococcus* sp. BUPNP1**	***R. rhodochrous* DSM43241**	***R. pyridinivorans* SB3094**	***R. aetherivorans* IcdP1**	***R. equi* 103S**	***R. jostii* RHA1**	***R. opacus* B4**	***R. erythropolis* PR4**	***R. fascian*s D188**
Accession No.	NERM00000000	LRRK0000000	CP006996	CP011341	FN563149	CP000431	AP011115	AP008957	CP015235
Aromatic compound degradation reported	4-NP (Sengupta and Saha, 2014)	–	MEK (Dueholm et al., 2014)	OCPs (Qu et al., 2015)	–	PAA, PCBs (Navarro-Llorens et al., 2005; Jones et al., 2013)	Benzene (Na et al., 2005)	4-NP, PAHs) (Sekine et al., 2006)	–
Genome size (Mb)	5.56	5.18	5.23	5.92	5.04	7.8	7.91	6.52	5.14
Plasmids	0	0	2	0	0	3	5	3	2
G + C content (%)	68.1	68.2	68	70.6	68.8	67.5	67.9	62.3	64.7
ANI (%) with BUPNP1	–	95.25	95.13	80.82	79.46	79.02	78.96	78.13	78.13
Genome nBLAST % similarity with BUPNP1	–	96	96	81	89	83	83	86	86
16S rRNA % similarity with BUPNP1	–	99.6	99	97	96	83	96	95	95
No. of genes (CDS)	5,033	4,803	4,818	5,388	4,649	7,262	7,287	6,092	4,808
Protein coding genes	4,963	4,585	4,580	5,020	4,540	6,894	6,983	5,949	4,689
No. rRNA genes	9	6	12	12	12	12	12	15	12
No. tRNA genes	54	54	55	54	52	50	50	54	46
No. other RNA genes	2	3	3	3	3	3	3	3	3
No. of pseudogenes	70	155	168	299	42	303	239	71	58
Average gene size (bp)	964.7	993.58	986.41	998.3	1005.6	968	984.97	967.47	980.36

*4-NP, 4-nitrophenol; MEK, methyl-ethyl-ketone; OCPs, organochlorine pesticides; PAA, phenyl acetic acid; PAHs, poly aromatic hydrocarbons; PCBs, poly chloro benzenes; ANI, average nucleotide identity*.

### BUPNP1 May Belong to a Novel *Rhodococcus* Species

Comparisons of ANI values (average nucleotide identity) between the genomes of BUPNP1 and other type strains of *Rhodococcus* spp. revealed the closest match to be *Rhodococcus rhodochrous* DSM43241 (95.25%) followed by *Rhodococcus pyridinivorans* SB3094 (95.13%). According to the standards proposed by Chun et al. (2018), ANI values above 96% can be taken to confirm two organisms belong to the same species, while ANI values below 95% indicate the two organisms belong to different species. Thus, while BUPNP1 is likely to represent a new species of *Rhodococcus*, further evidence is required to make such a proposal.

### The BUPNP1 Genome Is Enriched in Hypothetical Proteins

The relative abundance of each COG category (Table 2) was generally very similar in BUPNP1 compared to other 4-NP degrading strains of *Rhodococcus* [*R. imtechensis* (AJJH00000000), *R. jostii* RHA1 (CP000431), *R. opacus* B4 (AP011115), and *R. erythropolis* PR4 (AP008957)]. However, the BUPNP1 genome was significantly (*P* < 0.05) enriched in COG categories D (cell cycle control, cell division, chromosome partitioning) and L (replication, recombination and repair), and relatively impoverished in COG categories N (cell motility) and V (defense mechanisms). Interestingly, BUPNP1 was also significantly (*P* < 0.05) enriched in COG categories for hypothetical proteins, R (general function prediction only) and S (function unknown).

**Table 2:** Relative abundance (%) of proteins assigned to different COG categories for BUPNP1 and other *Rhodococci*.

**COG**	**BUPNP1**	***R. erythropolis* PR4**	***R. imtechensis* RKJ300**	***R. jostii* RHA1**	***R. opacus* B4**	
B	0.045	0.02	0.02	0.02	0.02	Chromatin structure and dynamics
C	7.88	6.47	7.71	8.17	8.16	Energy production and conversion
D	0.86	0.72	0.57	0.54	0.58	Cell cycle control and mitosis
E	7.39	8.57	7.58	8.42	8.36	Amino acid metabolism and transport
F	2.05	2.15	1.9	1.88	1.9	Nucleotide metabolism and transport
G	5.5	5.87	6.58	6.4	6.38	Carbohydrate metabolism and transport
H	4.41	5.89	5.81	6.22	6.08	Coenzyme metabolism
I	9.86	9.85	10.44	10.56	11.23	Lipid metabolism
J	3.72	4.28	3.58	3.31	3.43	Translation
K	9.66	10.42	10.51	10.06	10.06	Transcription
L	3.85	2.77	2.38	2.31	2.37	Replication and repair
M	3.07	4.21	3.65	3.31	4.08	Cell wall/membrane/envelop biogenesis
N	0.04	0.26	0.11	0.19	0.22	Cell motility
O	2.56	2.81	2.88	2.28	2.37	Post-translational modification, protein turnover, chaperone functions
P	5.45	7.1	5.7	5.35	5.61	Inorganic ion transport and metabolism
Q	6.83	5.42	6.79	6.84	6.85	Secondary structure
R	14.1	11.4	12.21	13.3	12.21	General functional prediction only
S	7.24	4.15	4.44	3.91	3.76	Function unknown
T	3.67	3.85	3.41	3.17	2.91	Signal transduction
U	0.64	0.53	0.44	0.35	0.42	Intracellular trafficking and secretion
V	1.07	2.68	2.58	2.15	2.18	Defense mechanisms

*Categories in which BUPNP1 had the greatest abundance are highlighted in gray*.

### The First Intermediate of 4-NP Degradation by BUPNP1 Is 4-NC

**Figure 1:**
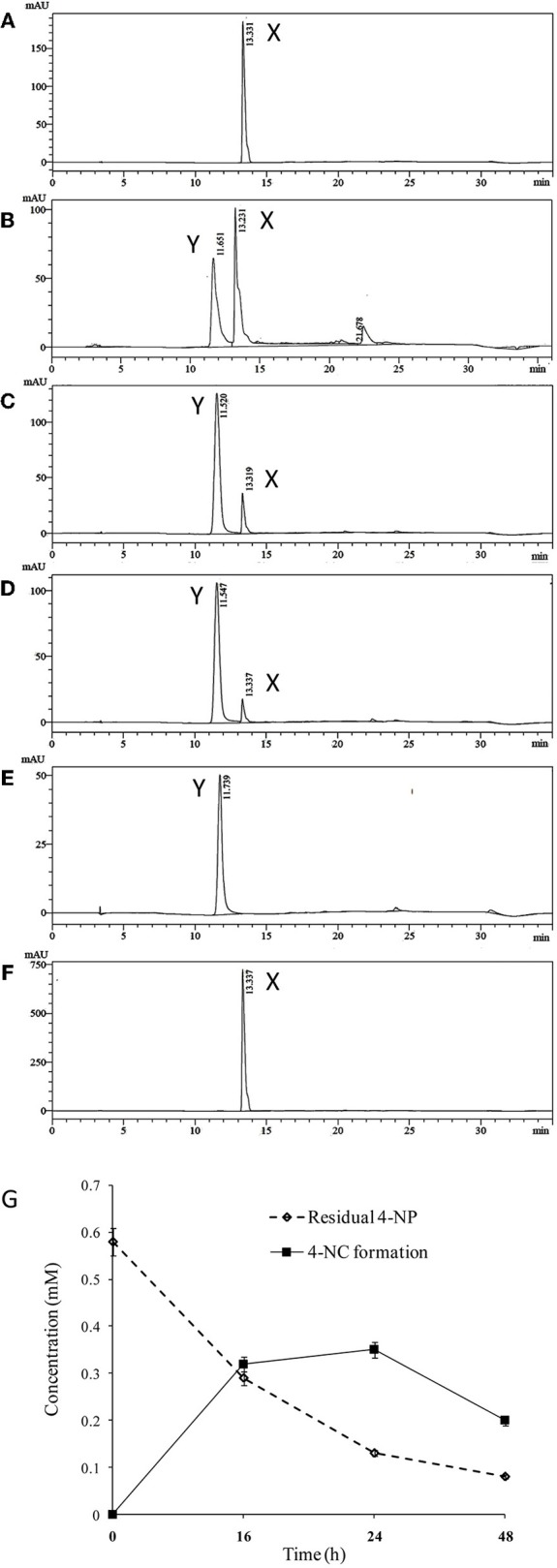
Degradation of 4-NP by strain BUPNP1. **(A–D)** HPLC chromatograms of metabolites extracted after 0, 24, 48, and 72 h, respectively of growth on 4-NP, **(E)** authentic standard of 4-NC, **(F)** authentic standard of 4-NP. X denotes 4-NP peaks, Y denotes 4-NC peaks. **(G)** Loss of 4-NP and accumulation of 4-NC over 48 h.

High performance liquid chromatography (HPLC) was used to quantify 4-NP degradation by cultures of BUPNP1 0, 24, 48, and 72 h after 4-NP addition. Peaks observed in culture supernatants (Figure 1) were identified as 4-NP and 4-NC, with retention times (RT) matching those of authenticated standards (RT of 13.3 and 11.5 min, respectively). After 72 h, 95% of added 4-NP had been degraded. Production of 4-NC was coincident with 4-NP degradation, peaking 48 h after 4-NP addition (Figure 1), suggesting that 4-NC is the first intermediate of 4-NP degradation. No other intermediates of 4-NP degradation (including BT) were observed by HPLC, even though both 4-NC and BT are able to support the growth of BUPNP1 as sole carbon sources (data not shown), possibly suggesting rapid conversion into non-aromatic metabolites. 4-NP degrading activity was also detected in enzyme assays (monitoring NADH consumption with a 4-NP substrate), with cell extracts taken from cultures 8–16 h post-inoculation, with a mean specific activity of 0.115 mmol min^−1^mg^−1^.

### The BUPNP1 Genome Contains a Cluster of Genes for Aromatic Compound Catabolism

Knowing that the first step in 4-NP metabolism by BUPNP1 is its conversion to 4-NC, candidate 4-NP monooxygenases were identified in the genome and the surrounding genomic region analyzed. Table 3 shows details of a cluster of 43 genes (BUPNP1_02455 to BUPNP1_02497) which are likely to be involved in the degradation of 4-NP and other aromatic compounds. BUPNP1_02486 and BUPNP1_02487 (*nphA2* and *nphA1*, respectively) are proposed to encode the reductase and oxygenase components of the putative BUPNP1 4-NP 2-monooxygenase (EC 1.14.13.29), and share substantial sequence similarity (76 and 85%, respectively) with the 4-NP-degrading genes of *Rhodococcus* sp. strain PN1 (Takeo et al., 2008).

**Table 3:** The *nph* gene cluster of BUPNP1.

**Locus tag**	**Strand**	**Gene**	**EC number**	**Protein name**	**Reaction catalyzed**	**FC 6 h**	**FC 12 h**
BUPNP1_02455	–	*scoB*	2.8.3.5	3-Oxoacid CoA-transferase	Succinyl-CoA + 3-oxoadipate > succinate + 3-oxoadipyl-CoA		1.65655
BUPNP1_02456	–	*scoA*	2.8.3.5	3-Oxoacid CoA-transferase	Succinyl-CoA + 3-oxoadipate > succinate + 3-oxoadipyl-CoA	−1.4708	
BUPNP1_02457	+	*pcaB*	5.5.1.2	3-Carboxy-cis,cis-muconate cycloisomerase	3-Carboxymuconolactone > 4-carboxymuconolactone	−1.75688	−2.62553
BUPNP1_02458	+	*catD pcaC*	4.1.1.44 3.1.1.24	4-Carboxymuconolactone decarboxylase	4-Carboxymuconolactone > 3-oxoadipate-enol-lactone		−1.0313
BUPNP1_02459	+	“*nphR3*”		IclR family regulator			−0.6726
BUPNP1_02460	+	*bktB*	2.3.1.16	Beta-ketothiolase	3-Oxoadipyl-CoA > succinyl-CoA + acetyl-CoA		
BUPNP1_02461	+	–		Hypothetical		−0.7456	0.79127
**BUPNP1_02462**	**–**	**“*****nphD*****”**	**1.3.1.–**	**NADPH-dependent oxidoreductase**	**Maleyl acetate** **>** **3-oxoadipate**		**0.74015**
BUPNP1_02463	–	–		Hypothetical			
BUPNP1_02464	–	–		Hypothetical			−2.15818
BUPNP1_02465	–	–		Hypothetical			
BUPNP1_02466	–	–	6.2.1.–	Aminoacid-acyl carrier protein ligase	Amino acid + [acyl-carrier protein] > aminoacyl-[acyl-carrier protein]		0.65156
BUPNP1_02467	–	–	1.3.99.–	Acyl-CoA dehydrogenase	Acyl-CoA > trans-2-enoyl-CoA		
BUPNP1_02468	–	–		Aminoacyl carrier protein			2.00598
BUPNP1_02469	–	–	2.7.8.7	Holo-acyl-carrier-protein synthase	CoA-[4′-phosphopantetheine] + apo-[acyl-carrier protein] > adenosine 3′,5′-bisphosphate + holo-[acyl-carrier protein]		−0.95415
BUPNP1_02470	–	*ndh*	1.6.99.3	NAD(P)-dependent reductase	NAD(P)H + acceptor < > NAD(P)+ + reduced acceptor		
BUPNP1_02471	+	*catM*		LysR family regulator			−0.91448
BUPNP1_02472	–	“*nphR2*”		PadR family regulator			1.58353
**BUPNP1_02473**	**+**	**“*****nphB2*****”**	**1.14.13.–**	**Aromatic ring-hydroxylating dioxygenase subunit alpha**	**4-Nitrocatechol** **>** **benzene 1,2,4-triol**	**−0.7456**	**−3.60422**
**BUPNP1_02474**	**+**	**“*****nphB1*****”**	**1.14.13.–**	**Aromatic ring-hydroxylating dioxygenase subunit beta**	**4-Nitrocatechol** **>** **benzene 1,2,4-triol**		**−3.41412**
BUPNP1_02475	+	–		Benzoate transporter			−2.45419
BUPNP1_02476	–	–		Transporter permease			−0.72507
BUPNP1_02477	+	–	3.1.1.–	Ester hydrolase+			1.36939
**BUPNP1_02478**	**+**	**“*****nphC*****”**	**1.13.11.37**	**Aromatic ring opening dioxygenase**	**Benzene 1,2,4-triol** **>** **maleyl acetate**		**0.66077**
BUPNP1_02479	–	–	3.1.1.–	Ester hydrolase			−1.27452
BUPNP1_02480	–	–	3.–.–.–	Hydrolase		−1.46609	−2.62852
BUPNP1_02481	–	*catC*	5.3.3.4	Muconolactone delta-isomerase	Muconolactone > 3-oxoadipate-enol-lactone		
BUPNP1_02482	–	*catB*	5.5.1.1	Muconate cycloisomerase 1	cis,cis-Muconate > muconolactone	−1.45262	
BUPNP1_02483	–	*catA*	1.13.11.1	Catechol 1,2-dioxygenase	catechol > cis,cis-muconate	−1.25922	0.80248
BUPNP1_02484	+	–		IclR family regulator			
BUPNP1_02485	–	“*nphR1*”		AraC family regulator		0.63538	
**BUPNP1_02486**	**+**	***nphA2***	**1.14.13.29**	**4-Nitrophenol 2-monooxygenase, reductase component**	**4-Nitrophenol** **>** **4-nitrocatechol**	**4.03225**	
**BUPNP1_02487**	**+**	***nphA1***	**1.14.13.29**	**4-Nitrophenol 2-monooxygenase, oxygenase component**	**4-Nitrophenol** **>** **4-nitrocatechol**	**4.26396**	
BUPNP1_02488	+	–		Hypothetical protein			
BUPNP1_02489	–	*pchR*		AraC family regulator			−0.9469
BUPNP1_02490	+	–	1.14.–.–	Cytochrome P450	Phenol > catechol	−2.09417	−2.16331
BUPNP1_02491	+	–	1.14.13.7	Phenol hydroxylase	Phenol > catechol	−2.16452	−2.09012
BUPNP1_02492	+	*cbdA*	1.14.12.13	(2-Halo)benzoate 1,2-dioxygenase, large subunit	(2-Halo)benzoate > catechol	1.88926	
BUPNP1_02493	+	*cbdB*	1.14.12.13	(2-Halo)benzoate 1,2-dioxygenase, small subunit	(2-Halo)benzoate > catechol	1.88019	
BUPNP1_02494	+	*cbdC benC*	1.18.1.3	(2-Halo)benzoate 1,2-dioxygenase electron transfer component	(2-Halo)benzoate > catechol	1.64361	
BUPNP1_02495	+	*benD*	1.3.1.25	1,2-Dihydroxycyclohexa-3,5-diene-1-carboxylate dehydrogenase	1,2-Dihydroxycyclohexa-3,5-diene-1-carboxylate > catechol	−1.62609	−0.90644
BUPNP1_02496	+	*benK*		Benzoate transporter		−2.72901	−1.54875
BUPNP1_02497	+	–		LuxR family regulator		−0.94986	−1.64115

**Figure 2:**
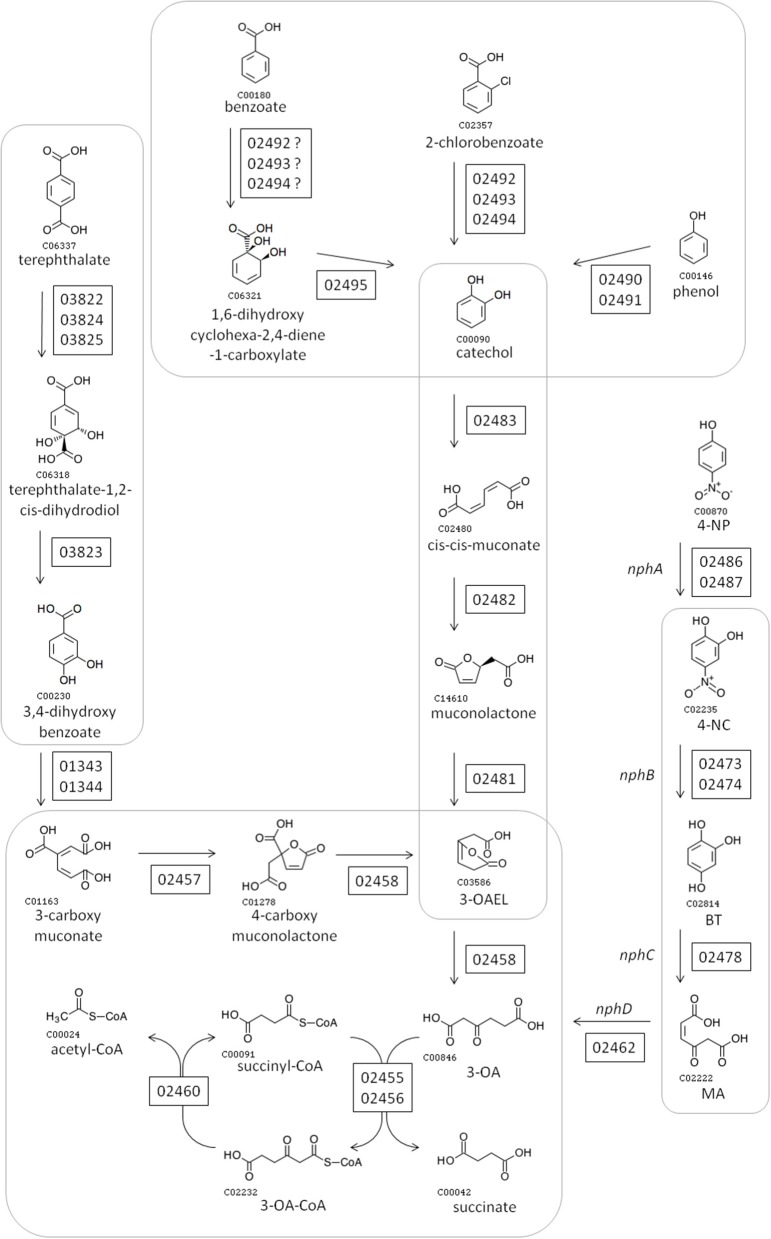
Proposed pathway for degradation of 4-NP and other aromatic compounds by BUPNP1. Numbers in rectangles near enzymatic steps represent BUPNP1 locus tag numbers for the genes encoding the enzyme(s) in question. Enzymatic reactions enclosed together in smooth-cornered rectangles can be considered sub-pathways, with successive conversions catalyzed by enzymes encoded in a putative operon. 3-OA, 3-oxoadipate; 4-NP 4-nitrophenol; 4-NC, 4-nitrocatechol; BT, benzene-1,2,4-triol; MA, maleyl acetate; 3-OAEL, 3-oxoadipate-enol-lactone.

*For each gene, genome organization, gene name, and protein product annotation are provided. For enzymes, the EC (Enzyme Commission) number is given, and the likely reaction catalyzed. For those genes exhibiting statistically significant differential expression when grown on 4-NP compared to glucose, the log_2_ fold-change (FC) in gene expression is presented, at 6 h and at 12 h (positive values indicate greater expression in 4-NP than in glucose). Rows are grouped together by shade (grays or white), where groups of rows represent genes likely functioning together to catalyze/regulate one of the sub-pathways proposed in Figure 2. Gene names are provided where a sequence ortholog has been unambiguously named in another organism. Those in quotes are names being proposed for this study. Genes encoding enzymes for the conversion of 4-NP into 3-OA are highlighted in bold*.

The gene cluster (designated here as “*nph*”) is composed of at least 14 transcriptional units, and includes gene encoding 27 metabolic enzymes, three metabolite transporters, seven regulatory proteins, five hypothetical proteins and one carrier protein. Most regulatory proteins and transporters were encoded as monocistronic units, with regulatory genes typically on the opposite strand to the transcriptional units whose expression they are most likely to regulate (assuming regulation of adjacent genes). Several transcriptional units encoded multiple enzymes that are known to act together to catalyze a pathway. For instance BUPNP1_02481 to BUPNP1_02483 encode the enzymes (CatC, CatB, and CatA, respectively) required for the conversion of catechol into 3-oxoadipate-enol-lactone (3-OAEL), while BUPNP1_02489 to BUPNP1_02497 encode the enzymes required to convert phenol, benzoate and halobenzoates into catechol.

Figure 2 shows the pathways encoded by the *nph* gene cluster. All enzymatic activities required for the complete conversion of 4-NP into AcCoA are encoded in the *nph* cluster, alongside enzymes for the synthesis/degradation of fatty acids, to/from AcCoA. 4-NP is proposed to be degraded into 4-NC (by “NphA” activity), 4-NC is converted into BT (by “NphB” activity), BT becomes MA (due to “NphC”), and “NphD” activity converts MA into 3-OA (Figure 2). This is the same pathway proposed for *R. imtechensis* RKJ300 by Ghosh et al. (2010). None of the BUPNP1 NphBCD proteins were found to be similar to any of the Nph or Nps proteins of *Rhodococcus* sp. strain PN1 (Takeo et al., 2008; Yamamoto et al., 2011), implying that acquisition of the enzymes catalyzing conversion of 4-NC into 3-OA was by horizontal transfer in BUPNP1 and/or PN1.

**Figure 3:**
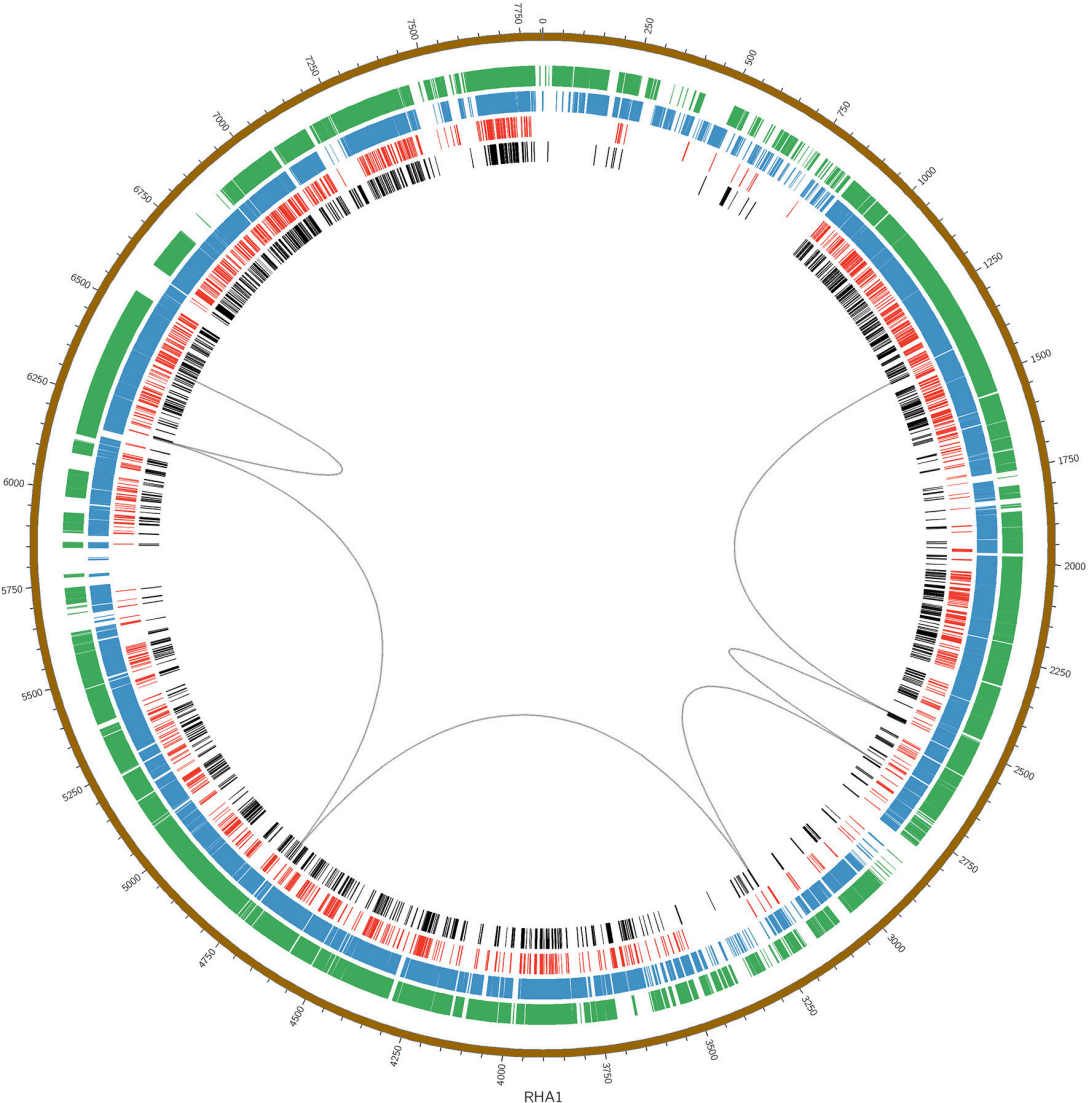
Pairwise alignments of the DNA sequence of BUPNP1 (black), *Rhodococcus erythropolis* strain BG43 (red), *Rhodococcus opacus* B4 (blue), and *Rhodococcus imtechensis* RKJ300 (green) to the reference genome of *Rhodococcus jostii* RHA1 (brown, outermost ring). Loops (black) connect the aligned regions of the *nph* cluster of BUPNP1 (genes BUPNP1_02455 to BUPNP1_02497), and show that the structure of the *nph* cluster is not conserved. Instead, the genes are distributed widely around the chromosomes of other *Rhodococcus* spp. strains.

The large *nph* gene cluster appears to be another novel feature of the BUPNP1 genome. Blocks of conserved DNA sequence (Figure 3) were identified across BUPNP1 and the four available genomes of 4-NP degrading *Rhodococcus* sp. strains (*Rhodococcus imtechensis* Strain RKJ300 (AJJH00000000), *Rhodococcus opacus* B4 (NC_012522), *Rhodococcus erythropolis* PR4 (NC_012490), and *Rhodococcus jostii* RHA1 (NC_008268). Although the *nph* gene cluster in BUPN1 is contained within a single contig, only 26% of sequence in this locus aligned to the RHA1 strain, and these alignments were found to be scattered widely across the genome. Not even microsynteny was observed. Not one of the polycistronic operons witin the *nph* gene cluster of BUPNP1 could be identified in the other 4-NP degrading *Rhodococci*. Pairwise alignments of the four genomes on to the BUPN1 gene cluster locus confirmed the low level of conserved gene synteny (data not shown).

### BUPNP1 Requires Time to Adapt to Growth on 4-NP

To ascertain which genes are induced/repressed during 4-NP degradation, a transcriptomic experiment was performed, comparing gene expression in cultures growing on 4-NP, with cultures growing on a non-aromatic carbon source. In order to identify suitable alternative non-aromatic carbon sources which could support the growth of BUPNP1, its carbohydrate utilization pattern was obtained using Vitek 2, an automated BIOLOG colourimetric assay system. BUPNP1 exhibited a catabolic versatility similar to that of other *Rhodococcus* sp., catabolising several sugars including glucose (Table 4).

**Table 4:** Vitek-2 substrate utilization profile (BIOLOG assay) of strain BUPNP1 (“+” is positive reaction while “–” is negative).

**Carbon source**	**Reaction**	**Carbon source**	**Reaction**	**Carbon source**	**Reaction**
L-Arabinose	+	Phosphoryl choline	–	Sodium gluconate	–
L-Lysine-arylamide	–	Pyruvate	+	Glycerol	+
L-Aspartate arylamide	–	Maltose	+	Salicin	–
Leucine-arylamide	+	D-Tagatose	–	Dulcitol	–
Phenylalanine arylamide	+	D-Trehalose	–	Inositol	+
L-Proline arylamide	+	Inulin	–	Malonate	–
Lactose	–	D-Glucose	+	D-Fructose	+
L-Pyrrolydonyl-arylamidase	–	D-Ribose	–	Adonitol	–
Cellobiose	–	Putrescine	–	Arabitol	–
Alanine arylamide	+	Palatinose	–	Erythiol	–
Tyrosine arylamide	+	L-Rhamnose	–	α-Methyl-D glucoside	–
Beta-N-acetyl glucosaminide	–	D-Sorbitol	+	Citrate	–
Ala-Phe-Pro arylamide	+	Esculin	–	Cellobiose	–
Cyclodextrin	–	Sucrose	+	Xylitol	–
D-galactose	–	Glycine Arylamidase	–	α-Methyl-D mannoside	–
Glycogen	–	D-Mannitol	–	ONPG (o-nitrophenyl β galactoside)	–
Myo-inositol	–	D-Mannose	–	Sorbose	–
Methyl-D-Glucopyranose	–	D-Melezitose	–	5,5′-Dithio-bis-(2-nitrobenzoic acid)	–
Methyl-D-Xylose	–	N-acetyl-D-glucosamine	–	Sodium succinate	+

BUPNP1 grew well when grown in minimal medium (MM) supplemented with either 0.5 mM 4-NP or MM with 0.55 mM glucose as the sole carbon and energy source. Growth of BUPNP1 on 4-NP and consumption of 4-NP were dependent on inoculum size (Supplemental File 3), thus cultures were inoculated to a high initial optical density. There was an extended lag phase in 4-NP-supplemented mineral medium culture, suggesting that growth of BUPNP1 on 4-NP required a period of adaptation, presumably for the induction of expression of 4-NP degradation genes which are unexpressed in the absence of 4-NP. Maximal growth was reached with both carbon sources after 36–48 h. For the transcriptome experiments described below, sampling times of 6 h and 12 h were chosen, as both cultures were growing at a similar rate at those time points, and had adapted to grow on their respective carbon sources.

### 4-NP Degradation Coincides With Loss of Lipid Inclusions

**Figure 4:**
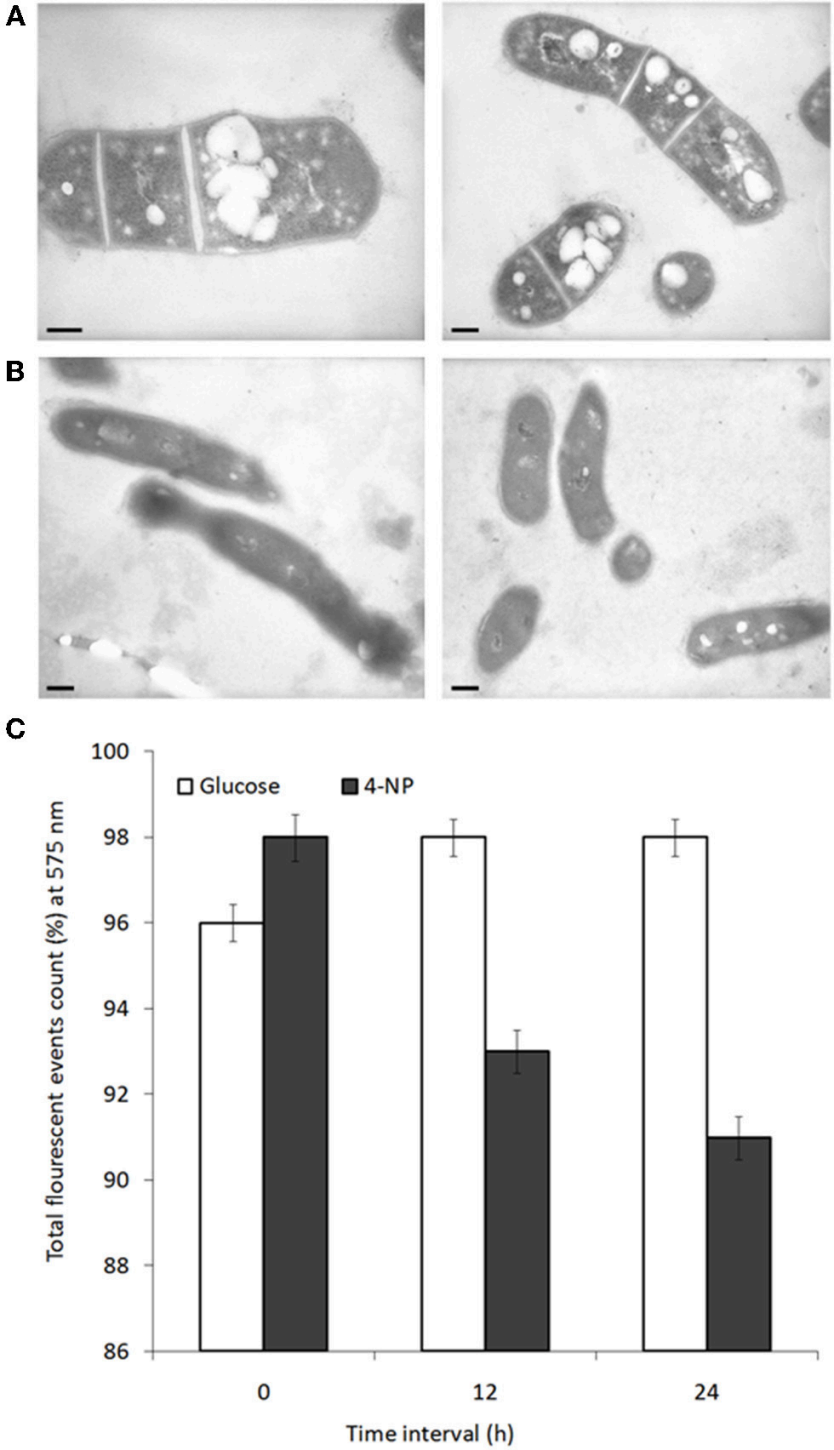
Lipid inclusions observed during growth on glucose are lost during growth on 4-NP. TEM images of cells grown for 12 h on **(A)** glucose, or **(B)** 4-NP. Bar = 200 nm. **(C)** Flow cytometric quantification of lipid content of cells pre-grown on glucose, stained with Nile red (total event counts % for emission at 575 nm) at 0, 12, and 24 h after shifting to growth on 4-NP.

Changes in cell physiology were noted depending on which carbon source BUPNP1 cells were cultured. Under TEM numerous large intracellular opaque droplets (inclusions) were observed in glucose-grown cells, but were absent from 4-NP-grown cells (Figure 4). Inclusions were confirmed to be lipid in nature through flow cytometric analysis with a non-specific lipid staining dye, Nile red, which emits fluorescence at 575 nm. Fluorescence intensity is proportional to the lipid content of cells, thus the gradual reduction of fluorescence in glucose-grown cells collected at 6 h, 12 h and 24 h after sub-culturing into 4-NP medium, indicates progressive loss of lipid content (Figure 4). Cells sorted on the basis of their fluorescence were further investigated by fluorescence microscopy confirming the absence of lipid inclusions in 4-NP-grown cells.

### Large Numbers of Genes Are Differentially Expressed When Degrading 4-NP

Three replicate cultures of four conditions (6 or 12 h growth in either minimal medium (MM) supplemented with 0.5 mM 4-NP, or MM with 0.55 mM of glucose as the sole carbon and energy source) were harvested and RNAseq performed. 5,672 gene transcripts were sequenced, with a mean length of 1,730 bp and median transcript length of 1,161 bp. After mapping reads to the BUPNP1 genome, relative expression levels of the genes were normalized for gene length and the resulting FPKM values (fragments per kilobase of transcript per million mapped reads) are provided in Supplemental File 4. Genes exhibiting differential expression (DE) when grown on glucose compared to 4-NP (at 6 and 12 h) are given in Supplemental File 5 (as log_2_ fold relative expression values with their probability score (*p*-value).

After 6 h of growth on 4-NP, 1,470 genes were differentially expressed when compared to growth on glucose, with 693 genes being up-regulated and 777 being down-regulated. After 12 h of growth 2,906 genes were significantly differentially expressed; 1,416 up-regulated and 1,490 down regulated. The proportion of hypothetical genes differentially expressed increased approximately from 6 to 11% between 6 and 12 h, suggesting that proteins with unknown functions are particularly involved in secondary/indirect aspects of the adaptation to growth on 4-NP.

### Most Genes of the *nph* Cluster Are Differentially Expressed During 4-NP Degradation

The expression profile (log_2_ fold change) for the genes of the *nph* cluster differentially expressed in glucose/4-NP are included in Table 3. After 6 h, the *nphA1* and *nphA2* genes were the most highly induced in 4-NP compared to glucose, but the *cbdABC* genes (whose gene products convert halobenzoate into catechol) were also up-regulated by 4-NP. By 12 h, both sets of genes were down-regulated again, so that there was no significant difference between their expression in 4-NP or glucose.

After 12 h of exposure to 4-NP, nine genes were induced relative to their expression in glucose, including BUPNP1_02478, the gene proposed for conversion of BT into maleyl acetate (MA), and BUPNP1_02462, proposed to convert MA into 3-oxoadipate (3-OA). This suggests that the conversion of 4-NP into 3-OA is not regulated as a single pathway at the transcriptional level. Earlier steps in the pathway are switched on by 6 h, while later parts of the pathway are instead switched on by 12 h, presumably as 4-NP has been converted into BT.

The remaining genes proposed to be part of the 4-NP to 3-OA degradation pathway (BUPNP1_02473 and BUPNP1_02474, for conversion of 4-NC into BT), were the most down-regulated genes at 12 h of growth in 4-NP compared to glucose (Table 3). This is consistent with above proposal, as it implies that by 12 h the BUPNP1_02473 and BUPNP1_02474 gene products had served their purpose.

## Discussion

### A Model for the Regulation of 4-NP Degradation by BUPNP1

**Figure 5:**
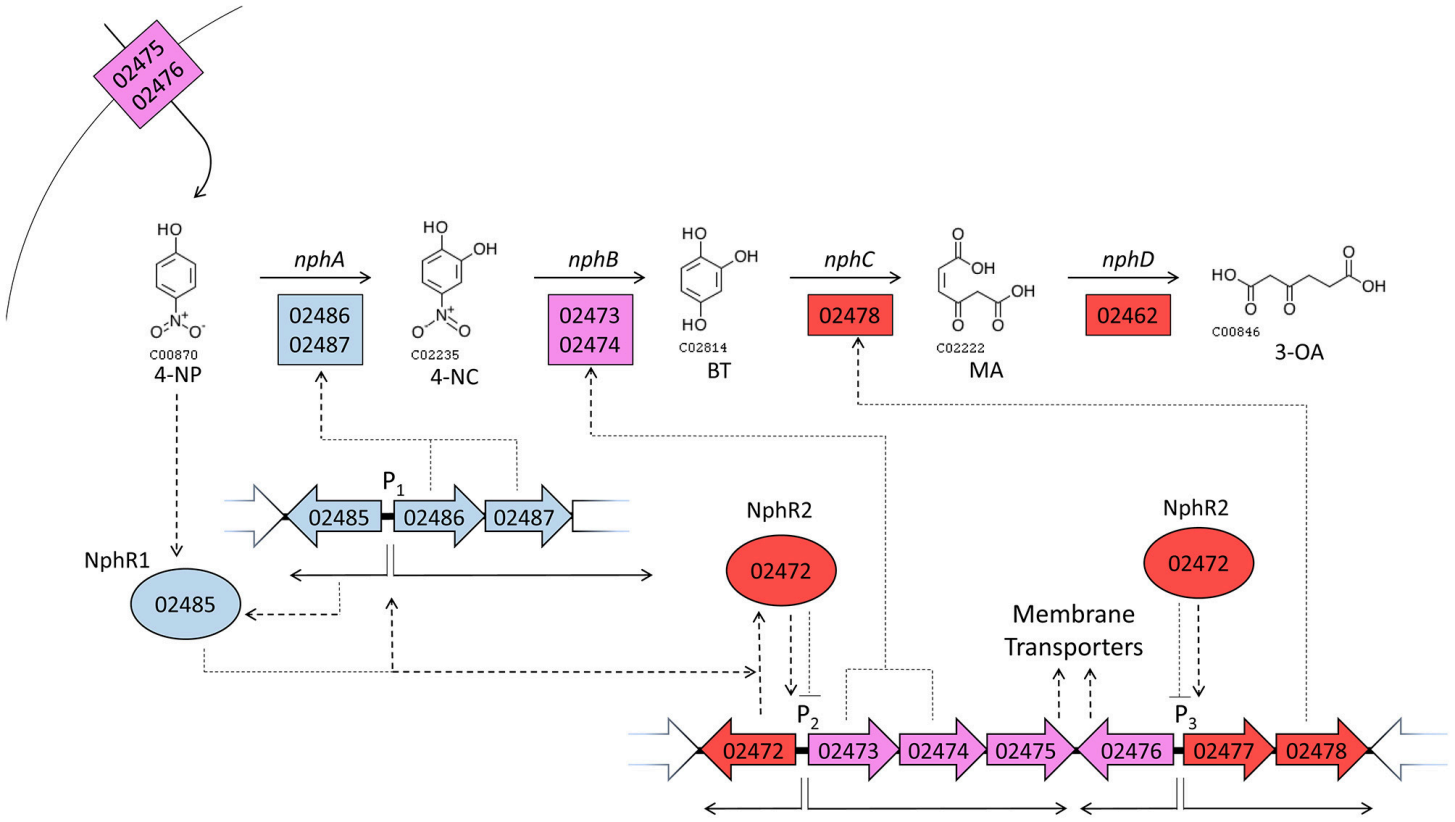
A model of gene regulatory events during growth on 4-NP. Large arrows with internal numbers represent genes with BUPNP1 locus tag numbers. Transcription from promoter regions (P) 1, 2 and 3 are shown as thin solid arrows, while translation and regulatory interactions are shown as dotted arrows. Positive relationships are pointed arrows, negative interactions have blunt arrowheads. Enzymes are shown as rectangles, regulatory proteins as ovals. The color of genes/proteins reflects their expression during growth in 4-NP relative to expression during growth in glucose. Blue genes/proteins are induced (positive FC in Table 3) at 6 h in 4-NP, but then uninduced by 12 h (insignificant FC). Purple genes/proteins are on at 6 h (insignificant FC), but repressed by 12 h (negative FC). Red genes/proteins are not induced at 6 h (insignificant FC), but are induced at 12 h (positive FC). Thus, blue and purple genes are expressed more at 6 h than at 12 h, while red genes are more highly expressed at 12 h than at 6 h. See discussion section A Model for the Regulation of 4-NP Degradation by BUPNP1 for a detailed description of the model.

A model is proposed (Figure 5) for the regulation of genes in the *nph* cluster, which takes into account their relative gene expression at 6 and 12 h. The model is predicated on the assumption that regulatory genes tend to regulate proximal genes, and tries to provide the most parsimonious explanation of all the available transcriptomic and genomic data. The model implicitly provides testable hypothesis regarding the functions and regulatory mechanisms of many genes governing 4-NP degradation.

When grown in glucose, genes encoding an aromatic compound transporter (BUPNP1_02475 and BUPNP1_02476) are expressed, as well as the genes for the conversion of 4-NC into BT (BUPNP1_02473 and BUPNP1_02474, “nphB1,” and “nphB2”). Upon exposure to 4-NP, uptake of 4-NP occurs and a 4-NP-sensitive AraC-family regulatory protein (BUPNP1_02485, “*nphR1*”) activates expression of itself, *nphA1* and *nphA2* (*nphA*), resulting in the conversion of 4-NP into 4-NC and then into BT.

By 12 h, the reduction of 4-NP concentrations has inactivated NphR1, switching off expression of the *nphRA2A1* operon. However, expression of a second regulatory gene (BUPNP1_02472, “*nphR2*”) has been activated, most likely by NphR1 (or possibly BT). NphR2 is a PadR-family repressor of its adjacent operon, switching off expression of *nphB* and the aromatic compound transporter. NphR2 may also be responsible for the induction of BUPNP1_02478 (*nphC*), whose expression causes the conversion of BT into MA. The genes responsible for the onwards conversion of MA into 3-OA (by BUPNP1_02462, *nphD*), 3-oxoadipyl-CoA, succinyl-CoA and acetyl-CoA (BUPNP1_02455 to BUPNP1_02462), are variously induced or repressed at 12 h, presumably as a consequence of regulation by the IclR family regulatory protein BUPNP1_02459 (“*nphR3*”).

### 4-NP Catabolism and Concurrent Fatty Acid Metabolism

Oleaginous strains of *Rhodococcus* sp. tend to store intracellular carbon-rich lipid in nitrogen limiting conditions, for instance while degrading non-nitrogen containing hydrocarbons, such as phenol, polyethylene and anthracene (Yoneda et al., 2016; Goswami et al., 2017; Gravouil et al., 2017). As 4-NP degradation provides both carbon and nitrogen for growth, the absence of nitrogen limitation is likely responsible for the observed lipid deterioration in BUPNP1 growing on 4-NP. Fatty acid degradation under such conditions could be increasing the C:N ratio of central metabolism for a more “balanced” efficient growth, or the resulting acetyl-CoA units could be being completely oxidized to provide extra energy for faster growth.

Surprisingly, a putative operon (BUPNP1_02463 to BUPNP1_02470) of eight genes for fatty acid synthesis/degradation proteins were found amongst the genes of the *nph* gene cluster (Table 3), suggesting that fatty acid metabolism and 4-NP degradation might be co-ordinately regulated. There was however inconclusive evidence of this in the gene expression dataset we generated, with just three of the genes being up-regulated and two down-regulated in 4-NP compared to glucose at 12 h.

The complete degradation of 4-NP is suggested to yield molar equivalents of succinate and acetyl-CoA (Figure 2). While acetyl-CoA can be completely oxidized by oxidative phosphorylation to provide energy equivalents, formation of succinate for the citric acid cycle is anaplerotic, thus 4-NP degradation may stimulate the citric acid cycle, allowing enhanced utilization of the acetyl-CoA from fatty acid or 4-NP oxidation. Alternatively succinate generation could allow increased siphoning of citric acid cycle intermediates for biosynthesis.

In theory, the catabolism of glucose by glycolysis and pyruvate dehydrogenase yields 2 acetyl-CoA, 2 ATP and 4 NADH. The pathway proposed here for 4-NP catabolism by BUPNP1 involves three oxygen-requiring steps (NphA, NphB, and NphC), and two steps involving redox co-factors. The NphA reaction (4-NP to 4-NC) oxidizes an NADH, while the NphD reaction (MA to 3-OA) generates an NAD(P)H, thus there is no net energy consumption or generation beyond the generation of a single acetyl-CoA per 4-NP. Thus, growth on 4-NP should be less than half as efficient as growth on glucose, yet growth rates are similar on the two substrates, presumably due to coincident fatty acid oxidation in 4-NP. It would be interesting to see how growth rate on 4-NP is affected by an absence of cellular lipid reserves—we would predict it would reduce more than 2-fold.

### Genomic Organization of the *nph* Gene Cluster

The *nph* cluster of 43 genes encodes mainly enzymes, but also encodes regulatory proteins, aromatic compound transporters and hypothetical proteins (Table 3). Genes encoding enzymes tended to be arranged into putative operons, with the encoded enzymatic activities catalyzing successive steps in pathways (Figure 2). The main exception being the 4-NP degradation pathway itself, where only the genes encoding the NphB and NphC reactions could be found within a putative operon. Another interesting feature of the *nph* cluster is that one gene (BUPNP1_02458) encoded a fusion protein of PcaA and CatD, thus catalyzing two reactions—the conversion of 4-carboxy muconolactone into 3-oxoadipate-enol-lactone (3-OAEL), and the conversion of 3-OAEL into 3-OA (Figure 2). CatD would normally be found encoded together with the genes for CatA, CatB, and CatD, however in this case the fusion protein is within a putative operon with genes converting 3-carboxy muconate via 3-OA into succinate and acetyl-CoA. A similar fusion protein has been described previously in *R. opacus* 1CP (Eulberg et al., 1998).

Unlike the genes for enzymes, regulators tended to be encoded as monocistronic transcripts, convergent with their likely target operons, mirroring the situation described in *R. opacus* SAO101 (Kitagawa et al., 2004). Of the seven regulators in the *nph* cluster, the central five are encoded in the opposite direction to both their adjacent genes (Table 3). Four of those regulators are encoded as two pairs of convergently encoded genes, including *nphR1* and *nphR2*, which exhibit further synteny, being encoded divergently from *nphA1A2* and *nphB1B2*, respectively (Figure 5). The seven regulatory proteins in the cluster belong to five different families of transcriptional regulators. NphR1 and PchR are AraC family members, NphR3 and BUPNP1_02484 are IclR family members, NphR2 is PadR family, CatM is LysR family and BUPNP1_02497 is LuxR family. Regulation of 4-NP degradation genes by AraC and LysR family regulators has been observed previously (Takeo et al., 2008; Yamamoto et al., 2011), however as homologous genes can be regulated by different regulatory protein family members in different organisms, family membership cannot be used to support or refute the model proposed above.

As well as the similarities in genome organization with *R. opacus* SAO101 mentioned above, the complete *nph* gene cluster is also present in the draft genome of *Rhodococcus rhodochrous* J3 (Genbank accession FXAV00000000.1), a lignin-degrading strain. Thus, mechanistic insights gained by studying 4-NP degradation in BUPNP1 may well be applicable across the *Rhodococcus* genus.

### Widespread Transcriptional Regulation in Response to Growth on 4-NP

In addition to the genes of the *nph* cluster there were many other genes around the genome which were significantly up- or down-regulated in 4-NP compared to glucose at 6 h or 12 h (Supplemental File 5). For instance, eight transcriptional regulators exhibited a log_2_ fold-change (FC) increase in gene expression in 4-NP compared to glucose of more than 2.0, as did six genes for transporter proteins, while 15 hypothetical genes had a FC of >3.5 in 4-NP compared to glucose.

Many aromatic compound metabolism enzymes from outside the 4-NP degradation pathway were also induced by 4-NP, including the *cbdABC* genes within the *nph* cluster, as mentioned earlier. Genes for gentisate dioxygenase (BUPNP1_4119), hydroxybutyrate dehydrogenase (BUPNP1_04091), protocatechuate 4,5-dioxygenase (BUPNP1_02577) and 2,3-dihydroxybiphenyl 1,2-dioxygenase (BUPNP1_02367) were also up-regulated with a FC >2.0 in 4-NP compared to glucose (Supplemental File 5). This may reflect co-occurance of the substrate molecules in the natural environment of BUPNP1, or overlapping specificity of their degradative pathways. Interestingly, two genes encoding enzymes of the citric acid cycle were mildly but significantly up-regulated at 12 h in 4-NP compared to glucose with a FC >0.5, namely succinate dehydrogenase (BUPNP1_01909) and isocitrate dehydrogenase (BUPNP1_03343).

Another gene cluster (*aro*) likely to be involved in aromatic compound degradation was also identified in the BUPNP1 genome (BUPNP1_03810 to BUPNP1_03834), which included the genes responsible for the conversion of terephthalate into 3,4-dihydroxy benzoate (BUPNP1_03822 to BUPNP1_03825) as shown in Figure 2. Other genes in the *aro* cluster include five regulatory proteins, three transport proteins and 12 enzymes, predicted variously to metabolize compounds including phenylacetone, 2-hydroxy-6-oxo-6-phenylhexa-2,4-dienoate (an intermediate of biphenyl degradation), 4-hydroxybenzoate, phenoxybenzoate and phthalate. Unfortunately, for most of the *aro* cluster enzymes, uncertainty in their annotation precludes us from proposing definitive pathways in which they might operate. Nevertheless, ten of the 25 *aro* cluster genes were significantly induced by 4-NP compared to glucose (another seven were significantly down-regulated), suggesting co-ordinated and integrated regulation and action of aromatic compound metabolism genes across the BUPNP1 genome.

## Conclusions

*Rhodococcus* sp. strain BUPNP1 can utilize the environmental pollutant 4-NP as its sole source of carbon and energy. 4-NC was identified as the first intermediate of 4-NP degradation in BUPNP1 and genes encoding the responsible 4-nitrophenol 2-monooxygenase (*nphA1* and *nphA2*) were identified by genome sequencing. The *nphA1* and *nphA2* genes were encoded within a cluster of 43 genes (the *nph* cluster) that together encoded enzymes for entire pathways for the degradation of 4-NP and other aromatics into metabolites of central metabolism (acetyl-CoA and succcinyl-CoA). Also encoded within the *nph* gene cluster were genes for fatty acid synthesis/degradation, and lipid bodies were observed to be consumed during growth on 4-NP but not during growth on glucose.

Investigation of the transcriptional response of BUPNP1 to growth on 4-NP revealed 4-NP-dependent regulation of the genes in the *nph* cluster. Patterns of expression were consistent with the genomic organization of the *nph* cluster genes—i.e., discrete transcriptional units responsible for sub-pathways within the larger metabolic network of aromatic compound degradation.

The data in this study have allowed the construction of a holistic testable model for 4-NP degradation by BUPNP1. The proposed model incorporates (i) the genes encoding the enzymes, transporters and regulators of aromatic compound degradation, (ii) transcriptional regulation of those genes, (iii) enzymatic activities of encoded enzymes, and (iv) the organization of those activities into pathways for the degradation of aromatic compounds, including 4-NP.

The model provides a framework for further investigations into the catalytic versatility and molecular adaptive responses of *Rhodococcus* sp. to growth on 4-NP, laying a foundation for rational engineering of strains for bioremediation of 4-NP and other aromatic pollutions.

### Nucleotide Sequence Accession Numbers

The genome sequence of *Rhodococcus* sp. BUPNP1 has been deposited in NCBI, GenBank under accession number NERM00000000.1. The raw reads have also been deposited at the NCBI FTP site via a Sequence Read Archive (SRA) submission under accession number SRR5483267. Reads from the transcriptome sequencing experiment have been deposited in the Sequence Read Archive under BioProjects PRJNA506436, PRJNA506437, PRJNA506434, and PRJNA504679.

## Author Contributions

KS and PS conceived the project. PS and DW supervised the research. KS performed the experiments and bioinformatics analyses. PL and MS assisted and directed bioinformatics analyses. KS and DW drafted the manuscript and all authors edited the manuscript.

### Conflict of Interest Statement

The authors declare that the research was conducted in the absence of any commercial or financial relationships that could be construed as a potential conflict of interest.

## Supplementary Material

Supplemental File 1 RAST annotation of CDS in the BUPNP1 genome.

Supplemental File 2 Comparison of the 4-NP biodegradation efficiencies reported for *Rhodococcus* strains.

Supplemental File 3 Inoculum dependence of BUPNP1 growth and 4-NP consumption.

Supplemental File 4 Relative expression of BUPNP1 genes after 6 and 12 h in 4-NP or glucose. FPKM values, normalized for gene length.

Supplemental File 5 Differentially expressed BUPNP1 genes in 4-NP vs. glucose, after 6 and 12 h.
