# Supplementary material for: Genome Sequencing and Comparative Transcriptomics Provide a Holistic View of 4-Nitrophenol Degradation and Concurrent Fatty Acid Catabolism by Rhodococcus sp. Strain BUPNP1
Source: Front Microbiol. 2019 Jan 4;9:3209. doi: 10.3389/fmicb.2018.03209 (PMC6328493; doi:10.3389/fmicb.2018.03209)
Supplement: Supplemental File 2 — Comparison of the 4-NP biodegradation efficiencies reported for Rhodococcus strains. [file Table_2.DOCX]

**Supplementary File 2:** 4-NP degradation efficiency of BUPNP1 and other strains of *Rhodocococcus* sp.

| **Name of strain**  **(**16S rRNA gene sequence accession no.) | **Degradation efficiency**  **(concentration, % 4-NP removal from medium)** | **Maximum 4-NP monooxygenase activity recorded** | **Isolation site** | **References** |
| --- | --- | --- | --- | --- |
| *Rhodococcus* sp. PN1 (AB044557)^#^ | 0.3 mM, Not determined | 6.6 ± 0.2 µmol min^-1^ mg^-1^ | Contaminated soil, Japan | Takeo *et al*., 2003 |
| *Rhodococcus opacus* SAO101 (AB032565)^#^ | 0.5 mM, Not determined | 26.4 ± 2.2 pmol min^-1^  µg^-1^ | Forest soil, Japan | Kitagawa *et al*., 2004 |
| *Rhodococcus* sp. CN6 (EU266492) ^#^ | 100 mg/L (0.72 mM), 100 % | Not determined | Industrial effluent sediment, China | Zhang *et al*., 2009 |
| *Rhodococcus imtechensis* RKJ300 (AY525785) ^#^ | 0.5 mM, 100 % | 1.4 µmol min^-1^ mg^-1^ | Pesticide-contaminated site, India | Ghosh *et al*.,  2010 |
| *Rhodococcus* sp. BUPNP1 (KF652059) | 0.5 mM, 95 % | 0.115 ± 0.1 mmol min^-1^ mg^-1^ | Landfill site, Burdwan, India | Sengupta and Saha, 2014 |

**References:**

Ghosh, A., M. Khurana, A. Chauhan, (2010) Degradation of 4-Nitrophenol; 2-chloro-4 nitrophenol and 2,4-Dinitrophenol by *Rhodococcus imtechensis* strain RKJ300, Environ. Sci. Technol. 44: 1069-1077.

Kitagawa, W., N. Kimura, Y. Kamagata, (2004) A novel *p*-nitrophenol degradation gene cluster from a gram-positive bacterium, *Rhodococcus opacus* SAO101, J. Bacteriol. 186: 4894-4902.

Sengupta, K., P. Saha, (2014) Identification of a newly isolated p-nitrophenol degrading strain characterized as *Rhodococcus* sp. BUPNP1, J. Environ. Res. Dev. 8: 431-436.

Takeo, M., T. Yasukawa, Y. Abe, S. Niihara, Y. Maeda, S. Negoro, (2003) Cloning and characterization of a 4-nitrophenol hydroxylase gene cluster from *Rhodococcus* sp. PN1. J. Biosci. Bioeng. 95: 139-45.

Zhang, J., Z. Sun, Y. Li, X. Peng, W. Li, Y. Yan, (2009) Biodegradation of *p*-nitrophenol by *Rhodococcus* sp. CN6 with high cell surface hydrophobicity. J. Hazard. Mater. 163: 723-8.
