# Supplementary material for: Genome Sequencing and Comparative Transcriptomics Provide a Holistic View of 4-Nitrophenol Degradation and Concurrent Fatty Acid Catabolism by Rhodococcus sp. Strain BUPNP1
Source: Front Microbiol. 2019 Jan 4;9:3209. doi: 10.3389/fmicb.2018.03209 (PMC6328493; doi:10.3389/fmicb.2018.03209)
Supplement: Supplemental File 3 — Inoculum dependence of BUPNP1 growth and 4-NP consumption. [file Table_3.DOCX]

**Supplemental File 3.** Inoculum size dependence of BUPNP1 growth and 4-NP consumption.

Maximal growth and consumption of 4-NP is observed with initial inoculation to an optical density (OD) at 600 nm of 0.6 or greater.

A similar phenomenon was described by Ramadan *et al.*, in 1990 (Inoculum size as a factor limiting success of inoculation for biodegradation. Appl Environ Microbiol. 56: 1392-6).
